# Associations between Age-Related Changes in the Core Vestibular Projection Pathway and Balance Ability: A Diffusion Tensor Imaging Study

**DOI:** 10.1155/2020/2825108

**Published:** 2020-02-11

**Authors:** Sang Seok Yeo, Jung Won Kwon, In Hee Cho

**Affiliations:** Department of Physical Therapy, College of Health Sciences, Dankook University, 119, Dandae-ro, Dongnam-gu, Cheonan-si, Chungnam 31116, Republic of Korea

## Abstract

**Objective:**

We investigated the changes of the vestibulospinal tract (VST) and parietoinsular vestibular cortex (PIVC) using diffusion tensor imaging (DTI) and relation to balance between old and young healthy adults.

**Methods:**

This study recruited eleven old adults (6 males, 5 females; mean age 63.36 ± 4.25 years) and 12 young adults (7 males, 5 females; mean age 28.42 ± 4.40 years). The lateral and medial VST and PIVC were reconstructed using DTI. Fractional anisotropy (FA), mean diffusivity (MD), and tract volume were measured. The six-minute walk test (6-MWT), the timed up and go test (TUG), and the Berg balance scale (BBS) were conducted. Spatiotemporal parameters during tandem gait and values of sway during one-leg standing using the wearable sensors were measured. All parameters between two groups were analyzed by the Mann-Whitney *U* test and independent *t*-test.

**Results:**

Statistically significant decrease in old adults was detected in the tract volume of lateral (*p* = 0.005) and medial VST (*p* = 0.005) and medial VST (*p* = 0.005) and medial VST (*p* = 0.005) and medial VST (*p* = 0.005) and medial VST (*p* = 0.005) and medial VST (*p* = 0.005) and medial VST (*p* = 0.005) and medial VST (*p* = 0.005) and medial VST (*p* = 0.005) and medial VST (*p* = 0.005) and medial VST (*p* = 0.005) and medial VST (*p* = 0.005) and medial VST (*p* = 0.005) and medial VST (*p* = 0.005) and medial VST (

**Conclusion:**

The results suggested that there was a relationship between DTI parameters in the vestibular neural pathway and balance according to aging.

## 1. Introduction

Increases in life expectancy and societal aging have highlighted functional problems experienced by the elderly during daily life and prompted studies on rehabilitation and improving quality of life [1, 2]. Balance is an important aspect of rehabilitation and the most basic factor in terms of ambulation and the risk of falls during everyday activities [1, 3, 4]. Balance maintains body posture and reduces fall risk [5, 6] and may be classified as static or dynamic. Balance control requires the complex integration of cognitive, nervous, and musculoskeletal systems [7, 8], and age-related changes in these systems are known to affect balance adversely and to increase fall risk [5, 9–11]. In 2018, Barela et al. investigated the sensitivity of passive motion in the ankle joint and the light touch and the relationship between the muscle strength and fall risk in the elderly. They reported that although sensitivity of passive motion deteriorated in lower limbs with age, the fingertip light touch could compensate for the somatosensory degradation of an old adult based on the fact that the body sway was reduced [10]. Hayes et al. reported motor learning differences between old and young groups in terms of sequence-specific task for 7 days and suggested that the old group needed more sequence training and practice [9].

Balance ability involves the visual, vestibular, and somatosensory systems [7]. Recently, studies on the relation between balance and the vestibular system have described age-related changes [12–14], and several studies suggested that age-associated reductions in vestibular function are caused by loss of neurons and vestibular hair cells in vestibular nuclei and diminished vestibuloocular reflex gain [13, 15]. The structures that mediate these vestibular functions are considered important, and structures of the central vestibular form are considered to be especially important [16]. The central vestibular structures that coordinate the balance system include, among other structures, the vestibulospinal tract (VST) and the parietoinsular vestibular cortex (PIVC). [11]. The VST is an extrapyramidal motor pathway to the spinal cord and controls balance [17, 18]. The VST processes visual information and decides which movements are required to maintain/control balance of an upright posture [19, 20]. Previous studies have reported that the PIVC is a core region of the vestibular cortex in the human brain [21, 22]. The PIVC is located in the posterior parietal operculum/retroinsular region and extends into posterior sections of the insular lobe [22, 23]. This area processes and integrates vestibular and somatosensory information when body and head positions change [24].

Diffusion tensor imaging (DTI) describes water diffusion patterns and thus provides a means of accessing the diffusion properties of white matter. DTI quantifies diffusion in multiple directions and enables functional connectivities and anatomical structures to be visualized [25–27]. Previous studies have reconstructed the vestibulospinal descending motor pathways and connectivity of PIVC in three dimensions [18, 28], and others have reported on the locations and functions of the VST and PIVC [17, 19, 22]. However, few studies assessed relations between age-related VST and PIVC connectivity changes.

In this study, we investigated the age-related changes of the vestibular pathway and the core vestibular cortex and their relations to balance using DTI in young and old healthy adults.

## 2. Material and Methods

### 2.1. Subjects

A summary of subject general characteristics is provided in Table 1. Of the 23 study subjects, 11 were assigned to the old group (6 males, 5 females; mean age 63.36 ± 4.25 years) and 12 to the young group (7 males, 5 females; mean age 28.42 ± 4.40 years). The subjects were recruited for this study at Yeungnam University Hospital. The study inclusion criteria were as follows: (1) no history of musculoskeletal or neurologic disease or cognitive problem and (2) no medical problem associated with the vestibular system. All subjects provided informed consent before undergoing DTI. DTI and balance ability assessments, which were performed after DTI, were conducted from 1 January 2019 to 1 March 2019. The study was approved beforehand by the institutional review board of Yeungnam University Hospital (YUH-18-05-028).

### 2.2. Diffusion Tensor Imaging

Acquisition of DTI data was performed using a 6-channel head coil on a 1.5 T Philips Gyroscan Intera (Philips, Best, the Netherlands) with single-shot echo-planar imaging. For each of the 32 noncollinear diffusion sensitizing gradients, 67 contiguous slices were collected parallel to the anterior commissure-posterior commissure line. The imaging parameters were as follows: acquisition matrix = 96 × 96, reconstructed matrix = 192 × 192, field of view = 240 × 240 mm^2^, TR = 10,726 ms, TE = 76 ms, parallel imaging reduction factor (SENSE factor) = 2, EPI factor = 49, *b* = 1000 s/mm^2^, NEX = 1, and a slice thickness of 2.5 mm with no gap (acquired voxel size, 1.3 × 1.3 × 2.5 mm^3^) [28, 29].

### 2.3. Probabilistic Fiber Tracking

The Oxford Centre for Functional Magnetic Resonance Imaging of the Brain (FMRIB) Software Library (FSL; http://www.fmrib.ox.ac.uk/fsl) was used to analyze diffusion-weighted imaging data. Head motion effect and image distortion due to eddy current were corrected using affine multiscale two-dimensional registration. Fiber tracking used a probabilistic image method based on a multifiber model and was performed in this study utilizing image routines implemented in FMRIB Diffusion (5000 streamline samples, 0.5 mm step lengths, and curvature thresholds = 0.2) [30].

**Figure 1:**
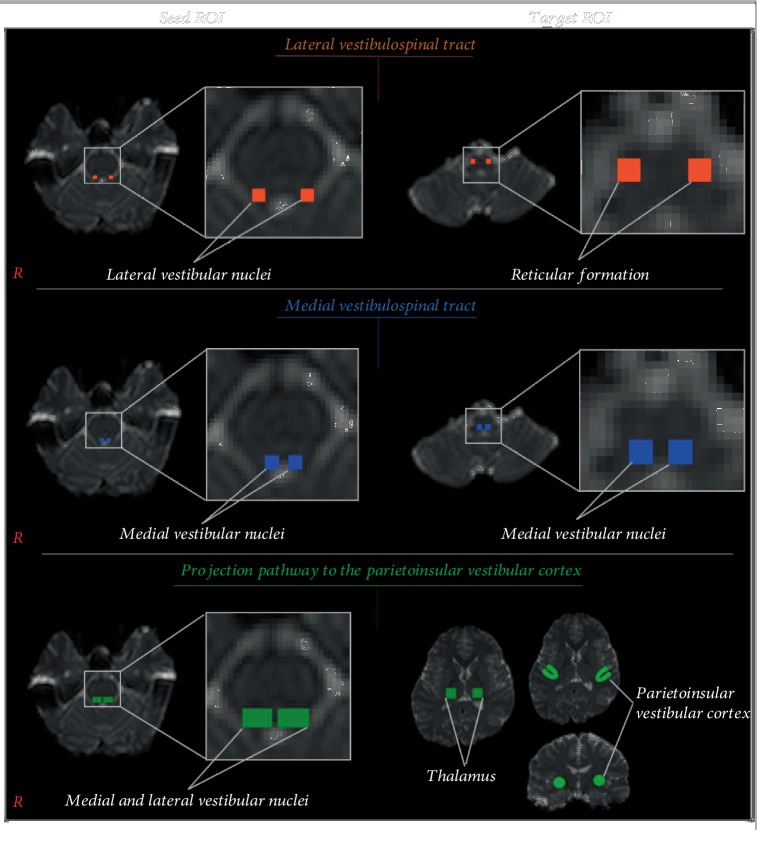
Seed regions of interest (ROIs) for lateral VST, medial VST, and the projection pathway to the PIVC were placed on lateral vestibular nuclei (orange) and medial vestibular nuclei (blue) and on vestibular nuclei (green) in the caudal portion of the pons. Target ROIs for VST and medial VSTs were placed on the reticular formation (orange) and the medial vestibular nuclei (blue) in the medulla, respectively. Two target ROIs on the projection pathway to the PIVC were placed on the thalamus and posterior parietal operculum (green).

The vestibular pathway and the core vestibular cortex were determined by selection of fibers passing through seed and one or two target regions of interest (ROIs). The seed ROI and target ROI of the vestibular projection pathway were determined as follows: lateral VST: seed ROI—the lateral vestibular nuclei (Deiters' nuclei) in the caudal portion of pons—and target ROI—the reticular formation at the level of the posterolateral medulla—and medial VST: seed ROI—the medial vestibular nuclei (Schwalbe's nuclei) in the pons—and target ROI—the medial vestibular nuclei in the medulla (Figure 1) [18]. To reconstruct the projection pathway to PIVC, the seed ROI was placed on the vestibular nuclei at the level of pons equivalent to Deiters' nuclei and Schwalbe's nuclei. Based on a previous study, two of the target ROI were placed on the posterior parietal operculum and thalamus (Figure 1) [31]. The 5000 samples were generated from the seed voxel, and the results were visualized at the threshold of 1 streamline through each voxel for analysis. We measured values of fractional anisotropy (FA), mean diffusivity (MD), and tract volume of the lateral and medial VST and the projection pathway to PIVC.

### 2.4. Measurements

#### 2.4.1. Tandem Gait

Spatiotemporal parameters were collected by LEGSys+ wearable sensor technology (BioSensics, Cambridge, Massachusetts, USA). Five wearable sensors (5.0 cm × 4.2 cm × 1.2 cm) were connected to a computer by Bluetooth and contained triaxial gyroscopes, accelerometers, and magnetometers [32]. Each sensor was attached by Velcro straps to the anterior surface of both shins 3 cm above the ankle, the anterior surface of both thigh 3 cm above the knee, and the low rear center of the posterior superior iliac spine (PSIS). Sampling frequency of sensors used in this study was 100 Hz. The subjects were asked to perform a tandem gait barefoot by placing the heel of the forward foot in front of toes of the back foot at each step without looking at their feet. Red cellophane tape was attached to the floor in a straight line to allow the participants to walk straight. The subjects performed the tandem gait along this red line on the floor and were instructed to lift their knees as high as 0.06 m during this procedure [33]. The subjects were also instructed to perform the tandem gait at a self-selected comfortable speed. Three trials were conducted for the tandem gait assessment.

#### 2.4.2. Functional Evaluation

The six-minute walk test (6-MWT) was performed to analyze motor control. Subjects were instructed to walk back and forth as quickly as possible on a 30 m walkway marked by two cones for 6 minutes. Total distances walked during three attempts were measured.

The timed up and go (TUG) test was performed to analyze functional mobility requiring dynamic balance. Subjects were instructed to sit with hips reaching the back of an armchair and on being given the signal “start” to stand up, to walk 3 m (marked by a cone) at a self-selected comfortable pace on a walkway, to turn around, to walk back to the armchair, and to sit down. The test was performed three times.

The Berg balance scale (BBS) was developed to measure fall risk. Testing involved 14 tasks, which were each scored from 0 to 4, that addressed various activities of daily living (e.g., standing, turning, and moving). Higher scores indicated better performances, and the test was also conducted three times.

#### 2.4.3. One-Leg Standing

Body sway was measured using the BalanSens system (BioSensics, Cambridge, Massachusetts, USA). One sensor (5.0 cm × 4.2 cm × 1.2 cm) was attached to the anterior surface of a shin 3 cm above the ankle of the side to be evaluated and the other to the low rear center of the PSIS. All sensors were connected to a computer via Bluetooth at a sampling frequency to 100 Hz. The subjects were asked to stand upright with arms by their sides and were instructed to lift the contralateral leg on hearing the signal “start” and maintain the state for 30 seconds [34]. Measurement was taken with eyes open (EO) and eyes closed (EC). Sways at the ipsilateral ankle, hip, and center of mass (COM) and the reciprocal compensatory index (RCI) were recorded. RCI represented as a function of correlation between movements of the hip and ankle joints. Testing was performed three times.

### 2.5. Statistical Analysis

The results were analyzed using SPSS ver. 20.0 (SPSS, Inc., Chicago, Illinois). The Shapiro-Wilk test was performed for the evaluation of the distribution of the variables. The Mann-Whitney *U* test determined the significances of differences between young and old healthy adults as follows: DTI parameters—MD of medial VST and FA and tract volume of PIVC, functional evaluation—BBS, and one-leg standing—hip sway and COM position in EO condition and ankle and hip sway, COM sway in the AP direction, and COM position in EC condition. The other results were analyzed between young and old healthy adults using the independent *t*-test based on the assumption of normality. Statistical significance was accepted for *p* values < 0.05.

## 3. Results

### 3.1. Diffusion Tensor Imaging

**Figure 2:**
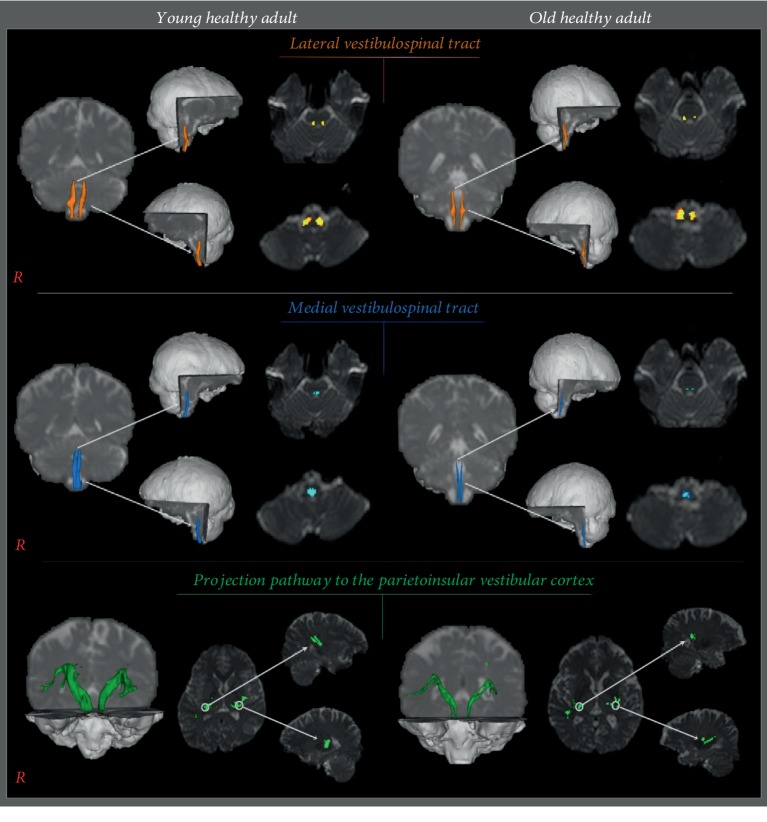
Reconstructed mean lateral VSTs (orange), medial VSTs (blue), and mean projection pathways to PIVC (green) for the two study groups.

Group DTI results were as follows. For lateral VSTs, mean FA (*p* = 0.044) and tract volume parameters were significantly smaller in the old group (*p* = 0.005), and for medial VSTs, MD was significantly higher (*p* = 0.001) and mean tract volume was significantly lower in the old group (*p* ≤ 0.001). Reconstructed projections to PIVC showed that mean tract volume was significantly lower in the old group (*p* = 0.020). However, group mean FA values for medial VSTs (*p* = 0.098) and PIVCs (*p* = 0.459) and mean MD values of lateral VSTs (*p* = 0.476) and PIVCs (*p* = 0.397) were not significantly different (Table 2, Figure 2).

### 3.2. Spatiotemporal Parameters

Spatiotemporal parameters for the two groups of adults during tandem gait are summarized in Table 3. Mean stride length (*p* = 0.001) and stride velocity (*p* ≤ 0.001) were significantly lower in the old group. However, mean stride time (*p* = 0.173) and cadence (*p* = 0.074) were not significantly different.

### 3.3. Functional Evaluations

Mean distance travelled in 6MWT was significantly less in the old group (*p* ≤ 0.001), and TUG was also significantly higher in the old group than in the young group (*p* ≤ 0.001). However, the mean BSS score was nonsignificantly lower in the old group (*p* = 0.296) (Table 4).

### 3.4. One-Leg Standing

#### 3.4.1. Eyes-Open Condition

The results of one-leg standing in the EO condition are provided in Table 5. With the exception of mean RCI in the mediolateral (ML) direction, which were nonsignificantly different (*p* = 0.301), all parameters except for mean RCI were significantly higher in the old adult (*p* < 0.05). The mean RCI in the anteroposterior (AP) direction was significantly lower in the old adult (*p* ≤ 0.001).

#### 3.4.2. Eyes-Closed Condition

Results obtained with EC during one-leg standing are provided in Table 5. Means sways in ankle (*p* = 0.031) and hip joints (*p* = 0.004) were significantly higher in the old group, as was COM sway in the AP direction (*p* = 0.014). However, mean COM sways in the ML direction (*p* = 0.395), COM positions (*p* = 0.074), and RCIs in all directions were not significantly different in the two groups (*p* > 0.05).

## 4. Discussion

In the present study, we reconstructed the vestibular projection pathway including the VST and the projection pathway to the PIVC as the core vestibular cortex. Tract volumes of medial and lateral VSTs and of PIVCs were found to be significantly lower in old healthy adults than in young healthy adults. In addition, mean FA of lateral VSTs and mean MDs of medial VST were significantly lower and higher, respectively, in the old group. However, no significant intergroup difference was observed between mean MDs of lateral VSTs or mean FAs of medial VSTs or between mean FAs and MDs of PIVCs. FA reflects the degree of white matter organization, that is, the directionalities and integrities of white matter microstructures, whereas MD provides a measure of water diffusion, which can change under pathologic conditions, such as in the presence of vasogenic edema or accumulated cellular debris resulting from axonal damage [26]. Tract volumes are determined by counting voxels contained by a neural pathway [35]. Therefore, observed reductions in FA values of lateral VSTs and in tract volumes of VSTs and of projection pathways to PIVCs in the old group suggest aging of white matter in the neural pathway. It has been well established that MD values are increased by the aging process and by neural injury [35–37]. Interestingly, unlike other studies, we observed a lower mean MD of the medial VST in the old group, which suggests a neural pathway compensatory mechanism for maintaining balance ability in the vestibular projection pathway including lateral VST and PIVC. Consequently, we assumed that lateral VST was most affected by aging compared to medial VST and projection pathways to PIVC.

We investigated balance function by assessing tandem gait, functional tasks, and one-leg standing performance. The summarized results of tandem gait analysis and of the functional evaluation were as follows: (1) significant differences were observed between the old and young groups in terms of stride length and stride velocity of spatiotemporal parameters during tandem gait, and (2) 6MWT distances and TUG times were significantly lower and higher, respectively, in the old group.

Regarding static balance, the one-leg standing test with EO showed (1) significantly higher sways of ankles and hips and of COMs in the ML and AP directions and in COM positions in the old group and (2) significantly lower RCIs in the AP direction, but not in the ML direction. The one-leg standing test with EC showed significantly higher sways of ankles and hips and of COMs in the AP direction in the old group. A previous study reported that the strategy shifted from the ankle joint to the hip joint because of the ankle muscle weakness and difficulty to generate the force at the ankle joint for balance control [38]. Therefore, these results are consistent with the increased hip sway angle of this current study in the old adults. However, COM sway in the ML direction, COM position, and RCI values were not significantly different in the two groups.

Although BBS scores were not significantly different, tandem gait and one-leg standing were significantly different in the two study groups, which concurs with previous reports [39, 40]. The authors of these studies also commented particular items of the BBS appearing to require high-level balance ability, and this suggestion is supported by our results. Therefore, reductions in tract volumes of the medial and lateral VSTs and of the projection pathway to the PIVC in the old group might be associated with reduced high-level balance ability (e.g., as indicated by tandem gait and one-leg standing stability). However, age-related changes in other DTI parameters of the projection pathway to the PIVC, such as those of the core vestibular cortex, were not found to adversely affect balance-related activities required for daily life in older adults.

With regard to fall risk, several previous studies have concluded that increased fall risk in the elderly is due to various neurological deficits [4, 5, 41–43]. Nakagawa et al. reported that gender did not influence balance, but that balance significantly decreased as age increased [5]. Iwamoto et al. showed that the elderly were more dependent on supporting structures for postural stability [43]. Many studies have been conducted to investigate age-related changes in brain structure volume [44–47]. Taki et al. investigated the relation between gray matter volume and age in healthy individuals and reported that cerebrovascular risk factors increased with age and that brain gray matter volume reduced [44]. Liu et al. found that white matter volume reduced with age and suggested that white matter volume reduces more rapidly than gray matter volume during the aging process [46]. In an MRI-based study on the relation between brain structure volumes and balance in the elderly, Makizako et al. compared the gray matter volumes of elderly fallers and nonfallers and found that fallers had smaller gray matter volumes, slower walking speeds, and shorter one-leg standing times [48].

Regarding DTI studies, some have examined the relation between changes in the brain structure and motor function in the elderly [49–51]. Serbruyns et al. examined associations between bimanual motor behavior and microstructural organizations of corpus callosum (CC) subregions in young and old adults and showed that microstructural organization of the CC correlated with bimanual performance in old adults and interestingly that finger manipulation deficits were associated with FA in the CC occipital subregion [49]. Fling et al. compared relations between callosal size and CC microstructural integrity and motor performance in young and elderly adults and observed that both were lower in the elderly cohort. They reported that elderly subjects showed bimanual control deficits, and the authors speculated that age-related declines of callosal size and CC integrity are related to reduced bimanual control [50]. On the other hand, Van Impe et al. in a study on the association between balance ability and retrogression of neural pathways in the context of postural control in the elderly demonstrated relations between age-related white matter structural changes and postural control; that is, they reported FA values of white matter pathways (e.g., corpus callosum, cingulum, fornix, and anterior thalamic radiation) and proprioceptive feedback was diminished in the elderly. As a result, these authors posited that white matter degeneration reduced postural control in the elderly [51]. However, no previous study has investigated the relation between age-related changes in the vestibular projection pathway or core vestibular cortex and motor deficits.

## 5. Conclusion

The present study was conducted to investigate age-related changes in the DTI parameters of the vestibular projection pathway, the projection pathway to the PIVC as the core vestibular cortex, and balance ability. The results obtained indicate changes in DTI parameters in the vestibular neural pathway and are associated with age-related reductions in balance ability. This is the first study to investigate the association between age-related changes in DTI parameters in vestibular neural pathways and changes in balance ability. The study provides basic data that could be used to plan exercise programs designed to mitigate fall risk. However, the study has several limitations. First, the small number of subjects recruited limits the generalizability of our findings. Second, it was difficult to locate ROIs precisely because of the diminutive sizes of vestibular nuclei. Third, we did not consider neuroplasticity because the subjects of this study were healthy adults. Therefore, we suggest that future studies be undertaken in patients with vestibular deficits or central vestibular disorders.

## Figures and Tables

**Table 1:** Demographic data of the young and old healthy adults.

	Old (*n* = 11)	Young (*n* = 12)
Age (years)	63.36 (4.25)	28.42 (4.40)
Gender (male/female)	6/5	7/5
Weight (kg)	62.82 (9.68)	66.42 (12.41)
Height (cm)	163.27 (5.85)	170.92 (7.91)

Values represent mean (±standard deviation).

**Table 2:** Comparison of DTI parameters in lateral VST and medial VST and PIVC between young and old healthy adults.

	Lateral VST			Medial VST			PIVC		
	FA	MD	Tract volume	FA	MD	Tract volume	FA	MD	Tract volume
Old	0.35 (0.05)	1.27 (0.26)	334.27 (135.47)	0.44 (0.05)	0.92 (0.12)	255.09 (118.89)	0.40 (0.05)	0.85 (0.06)	1862.86 (1971.90)
Young	0.38 (0.04)	1.22 (0.17)	454.833 (142.54)	0.41 (0.05)	1.03 (0.12)	402.96 (123.09)	0.40 (0.03)	0.84 (0.03)	2816.00 (1752.75)
*p*	0.044^∗^	0.476	0.005^∗^	0.098	0.001^∗^	0.000^∗^	0.459	0.397	0.020^∗^

Values represent mean (±standard deviation). DTI: diffusion tensor imaging; VST: vestibulospinal tract; PIVC: parietoinsular vestibular cortex; FA: fractional anisotropy; MD: mean diffusivity. ^∗^*p* < 0.05.

**Table 3:** Comparison of spatiotemporal parameters between young and old healthy adults during tandem gait.

	Stride length (m)	Stride time (s)	Stride velocity (m/s)	Cadence (steps/min)
Old	0.96 (0.11)	1.18 (0.12)	0.83 (0.14)	102.86 (9.77)
Young	1.15 (0.13)	1.12 (0.92)	1.05 (0.10)	109.54 (6.69)
*p*	0.001^∗^	0.173	0.000^∗^	0.074

Values represent mean (±standard deviation). ^∗^*p* < 0.05.

**Table 4:** Comparison of the results of functional evaluation between young and old healthy adults.

	6MWT (m)	TUG (s)	BBS (score)
Old	400.91 (24.27)	7.99 (0.52)	55.91 (0.30)
Young	565.83 (58.42)	6.85 (0.42)	56.00 (0.00)
*p*	0.000^∗^	0.000^∗^	0.296

Values represent mean (±standard deviation). 6-MWT: six-minute walk test; TUG: timed up and go; BBS: Berg balance scale. ^∗^*p* < 0.05.

**Table 5:** Comparison of the values of sway between young and old healthy adults during one-leg standing with eyes-open and eye-closed conditions.

	Anklesway (deg^2^)	Hipsway (deg^2^)	COM			RCI	
			ML (cm)	AP (cm)	Position (cm^2^)	ML	AP
Eyes open							
Old	57.43(32.60)	53.11(66.50)	1.82(0.70)	3.38(1.25)	6.55(4.41)	0.50(1.12)	0.50(0.10)
Young	12.38(5.40)	11.64(6.87)	1.23(0.24)	1.85(0.52)	2.29(0.89)	0.56(0.15)	0.73(0.13)
*p*	0.000^∗^	0.001^∗^	0.011^∗^	0.001^∗^	0.005^∗^	0.301	0.000^∗^
Eyes close							
Old	81.26(59.40)	120.53(64.99)	2.42(0.78)	4.37(1.69)	10.43(6.31)	0.69(0.12)	0.60(0.14)
Young	56.45(91.34)	42.67(47.56)	2.02(1.34)	3.19(2.57)	7.63(10.27)	0.67(0.08)	0.62(0.11)
*p*	0.031^∗^	0.004^∗^	0.395	0.014^∗^	0.074	0.769	0.704

Values represent mean (±standard deviation). COM: center of mass; RCI: reciprocal compensatory index; ML: mediolateral direction; AP: anteroposterior direction. ^∗^*p* < 0.05.

## Data Availability

Our data are available from the corresponding author upon request..
